# Clinical aspects and predictors of mortality of *Pseudomonas aeruginosa *pneumonia in a cohort of critically ill patients

**DOI:** 10.1186/cc9604

**Published:** 2011-03-11

**Authors:** G De Pascale, F Antonicelli, R Maviglia, A Cataldo, R Festa, F Idone, EM Trecarichi, MA Pennisi, M Tumbarello, M Antonelli

**Affiliations:** 1 Department of Anesthesiology and Intensive Care, Sacro Cuore Catholic University, Rome, Italy; 2 Institute of Infectious Diseases, Sacro Cuore Catholic University, Rome, Italy

## Introduction

*Pseudomonas aeruginosa *(PA) pneumonia (PN) represents a serious complication of long-term hospitalization [1]. The aim of our study is to analyze the clinical characteristics and predictors of mortality of PAPN in critically ill patients.

## Methods

All patients admitted to the 18-bed ICU of our university hospital between 1 January 2009 and 30 June 2010, affected by PAPN, were retrospectively enrolled in a cohort study.

## Results

Over the study period 1,109 patients were admitted and 322 bacterial PN were diagnosed. Sixty-five PAPN occurred: 52 ICU-acquired (ICUa) and 13 non-ICU-acquired (nICUa). Patients were mainly admitted because of a medical condition (71%), with a median length of ICU stay of 29.2 ± 27.6 days. The median SAPS II and SOFA scores were 40 ± 13.5 and 6.2 ± 3. A total of 35.4% of PA isolated were multidrug-resistant (MDR), 49.2% of patients with PAPN received a >24 hour delayed adequate antimicrobial treatment (DAAT >24 hours) and 57% received an anti-pseudomonas combination therapy; 25 patients (38.5%) died in the ICU. Comparing patients with ICUaPN with those with nICUaPN, the former group were younger (*P *< 0.01), with a longer length of ICU stay (*P *< 0.01), more frequently admitted for a traumatic reason (*P *= 0.02) and presented less severe SAPS II (*P *< 0.05). The independent risk factors associated with ICU mortality are listed in Table 1.

**Table 1:** Chronic renal failure (CRF)

	*P *value	OR
CRF	0.01	12.2 (1.6 to 91)
MDR PA	0.01	5.9 (1.4 to 25.6)
DAAT >24 hours	0.01	5.8 (1.4 to 23.6)
SAPS II score	0.01	1.1 (1.01 to 1.13

## Conclusions

PA has appeared as a relevant respiratory pathogen in our cohort of critically ill patients, either in ICU or pre/ICU settings. Patients' (baseline clinical condition), PA (MDR) and physicians' (DAAT >24 hours) related factors can influence the outcome of PN. The knowledge of local bacterial epidemiology and the prompt use of an anti-pseudomonas empiric treatment in patients with recognized PA risk factors could improve the outcome of severe MDR PAPN.
